# Widespread Distribution and Functional Specificity of the Copper Importer CcoA: Distinct Cu Uptake Routes for Bacterial Cytochrome *c* Oxidases

**DOI:** 10.1128/mBio.00065-18

**Published:** 2018-02-27

**Authors:** Bahia Khalfaoui-Hassani, Hongjiang Wu, Crysten E. Blaby-Haas, Yang Zhang, Federica Sandri, Andreia F. Verissimo, Hans-Georg Koch, Fevzi Daldal

**Affiliations:** a Department of Biology, University of Pennsylvania, Philadelphia, Pennsylvania, USA; b Biology Department, Brookhaven National Laboratory, Upton, New York, USA; c Institut für Biochemie und Molekularbiologie, ZMBZ, Faculty of Medicine, Albert-Ludwigs-Universität, Freiburg, Germany; University of Chicago

**Keywords:** CcoA, cytochrome, copper homeostasis, copper transport, copper uptake, cytochrome biogenesis, cytochrome *c* oxidase

## Abstract

Cytochrome *c* oxidases are members of the heme-copper oxidase superfamily. These enzymes have different subunits, cofactors, and primary electron acceptors, yet they all contain identical heme-copper (Cu_B_) binuclear centers within their catalytic subunits. The uptake and delivery pathways of the Cu_B_ atom incorporated into this active site, where oxygen is reduced to water, are not well understood. Our previous work with the facultative phototrophic bacterium *Rhodobacter capsulatus* indicated that the copper atom needed for the Cu_B_ site of *cbb*_3_-type cytochrome *c* oxidase (*cbb*_3_-Cox) is imported to the cytoplasm by a major facilitator superfamily-type transporter, CcoA. In this study, a comparative genomic analysis of CcoA orthologs in alphaproteobacterial genomes showed that CcoA is widespread among organisms and frequently co-occurs with cytochrome *c* oxidases. To define the specificity of CcoA activity, we investigated its function in *Rhodobacter sphaeroides*, a close relative of *R. capsulatus* that contains both *cbb*_3_- and *aa*_3_-Cox. Phenotypic, genetic, and biochemical characterization of mutants lacking CcoA showed that in its absence, or even in the presence of its bypass suppressors, only the production of *cbb*_3_-Cox and not that of *aa*_3_-Cox was affected. We therefore concluded that CcoA is dedicated solely to *cbb*_3_-Cox biogenesis, establishing that distinct copper uptake systems provide the Cu_B_ atoms to the catalytic sites of these two similar cytochrome *c* oxidases. These findings illustrate the large variety of strategies that organisms employ to ensure homeostasis and fine control of copper trafficking and delivery to the target cuproproteins under different physiological conditions.

## IMPORTANCE

The *cbb*_3_- and *aa*_3_-type cytochrome *c* oxidases belong to the widespread heme-copper oxidase superfamily. They are membrane-integral cuproproteins that catalyze oxygen reduction to water under hypoxic and normoxic growth conditions. These enzymes diverge in terms of subunit and cofactor composition, yet they all share a conserved heme-copper binuclear site within their catalytic subunit. In this study, we show that the copper atoms of the catalytic center of two similar cytochrome *c* oxidases from this superfamily are provided by different copper uptake systems during their biogenesis. This finding illustrates different strategies by which organisms fine-tune the trafficking of copper, which is an essential but toxic micronutrient.

## INTRODUCTION

Copper (Cu) is an important micronutrient required for the survival of virtually all living organisms, as numerous cellular processes depend on cuproproteins (1, 2). At high concentrations, Cu is extremely toxic for cells and can cause severe oxidative damage by competing with other divalent metal cations (e.g., iron) or by triggering the Fenton reaction (3, 4). Indeed, both Cu deficiency and excess cause serious human disorders, including Menkes, Wilson’s, and Alzheimer’s diseases (5–7). Therefore, Cu homeostasis is crucial for organisms, and cells tightly control their Cu content, from its uptake to its incorporation into cuproproteins (8, 9).

Heme-copper oxidases (HCOs) are major Cu-containing enzymes located in the cytoplasmic membranes of bacteria and archaea and in the mitochondrial inner membranes (10, 11). They are widespread among all domains of life as key components of the respiratory electron transport chain, catalyzing the reduction of oxygen to water while pumping protons across the membrane. HCOs are classified in three major families (A, B, and C) based on conserved residues forming the proton pathways within their catalytic subunit I (12). Although they differ in the number of subunits and cofactor composition, they all contain a conserved catalytic subunit carrying a low-spin heme and a heterobinuclear metal center composed of a Cu atom (referred to as Cu_B_) and a high-spin heme. The type A *aa*_3_-type cytochrome (cyt) *c* oxidases (*aa*_3_-Cox) are present in mitochondria and widespread in bacteria and archaea (13, 14). Two of the subunits of *aa*_3_-Cox harbor all of the cofactors required for catalysis. Subunit I (Cox1) is the conserved membrane integral catalytic subunit, which contains a low-spin heme *a* and a heterobinuclear center composed of a high-spin heme *a* (heme *a*_3_) and a Cu_B_ atom. Subunit II (Cox2) contains a homobinuclear Cu center (Cu_A_) that receives electrons from a cyt *c* donor. Depending on the species, the *aa*_3_-Cox enzymes may contain additional subunits with no cofactors. Biogenesis of the Cu centers of mitochondrial *aa*_3_-Cox requires plasma membrane-integral Cu transporters (known as Ctr) that import Cu into the cytoplasm and multiple chaperones (11, 15, 16). In yeast mitochondria, Cu is imported into the mitochondrial matrix before being inserted into *aa*_3_-Cox (17). The Cu chaperone Cox17 conveys the Cu in the mitochondrial intermembrane space to Sco1/Sco2 proteins for incorporation into the Cu_A_ center in Cox2 or to Cox11 for assembly of the Cu_B_ center in Cox1 subunits, respectively (18–21). In bacteria, periplasmic chaperones (e.g., PCu_A_C-like [22]) act as functional homologues of mitochondrial Cox17 and, together with the homologues of Sco1/Sco2 (SenC [23] or PrrC [24]), deliver Cu to the Cu_A_ center of *aa*_3_-Cox (22). Similarly, Cox11 homologues are required for the insertion of Cu_B_ into bacterial *aa*_3_-Cox, but its source of Cu remains unknown (24, 25).

Class C HCOs are *cbb*_3_-type cyt *c* oxidases (*cbb*_3_-Cox) that are found only in bacteria (26). They are the most divergent type of HCO and differ from class A *aa*_3_-Cox by containing three functional subunits (instead of two) and different cofactors (27). CcoN (subunit I) is the main catalytic subunit, which is the functional analogue of Cox1. It is an integral membrane protein and contains a low-spin heme *b* and a heterobinuclear center composed of a high-spin heme *b* (heme *b*_3_) coupled to the Cu_B_ atom. *cbb*_3_-Cox has no structural homologue of Cox2 or Cu_A_ center (27). Instead, it contains a dihemic cyt *c* subunit (CcoP), acting as the primary electron acceptor of *cbb*_3_-Cox. CcoP transfers the electrons via the monohemic cyt *c* subunit (CcoO) to the low-spin heme *b* and finally to the binuclear center heme *b*_3_-Cu_B_ of the CcoN subunit (13, 28). Cu_B_ atom incorporation into CcoN requires several transporters and chaperones (27). The P_1B_-type ATPase CcoI (29) (also known as CopA2 [30] or CtpA [31]), which is similar to the Cu-detoxifying transporter CopA (32) (or CopA1 [30]), is a Cu exporter located in the cytoplasmic membrane and is required for *cbb*_3_-Cox biogenesis. The fate of the Cu exported by CcoI is currently unclear. Possibly, it can be delivered either directly to the catalytic center of CcoN or to other periplasmic Cu chaperones. At low Cu availability, the Cu chaperones SenC (in *R. capsulatus* [23]) and PrrC (in *R. sphaeroides* [24]), which are homologues of mitochondrial Sco1/Sco2, and their interacting partners PccA (of *R. capsulatus* [33]) and PCu_A_C (of *R. sphaeroides* [24]) are needed for *cbb*_3_-Cox biogenesis. Finally, a member of the major facilitator superfamily (MFS) of transporters, CcoA, is a Cu importer that is required for assembly of the Cu_B_ center of *cbb*_3_-Cox (32, 34, 35). *R. capsulatus* mutants lacking CcoA are impaired for Cu uptake and contain a very small amount of *cbb*_3_-Cox (34). This importer is the first MFS member involved in metal transport and defines a new family of “copper uptake porters” (2.A.1.81) among the MFS-type transporters (http://www.tcdb.org/) (36). Recent studies showed that conserved Met and His residues of CcoA are important for its function, possibly acting as metal ligands (37). It is noteworthy that both the Cu importer CcoA and the Cu exporter CcoI are required to incorporate the Cu_B_ center into CcoN, implying trafficking of Cu across the cytoplasmic membrane during *cbb*_3_-Cox biogenesis (32).

In this study, we conducted comparative genomic analyses of CcoA orthologs in alphaproteobacterial genomes. This search revealed a higher degree of co-occurrence of CcoA with *cbb*_3_-Cox than with *aa*_3_-Cox, suggesting that CcoA activity is specific to class C HCOs. To test this hypothesis, we investigated the function of CcoA in *R. sphaeroides*, a close relative of *R. capsulatus*, which contains both functional *cbb*_3_- and *aa*_3_-Cox with identical heme-Cu_B_ binuclear centers, belonging to different HCO families. Upon the identification of *R. sphaeroides ccoA* (RSP_2726), appropriate mutants were constructed and their physiological and biochemical properties were characterized. We also identified bypass suppressors of Δ*ccoA* mutants in *R. sphaeroides copA* (RSP_2890) that restored *cbb*_3_-Cox activity at the expense of increased Cu^2+^ sensitivity. This study showed that CcoA is specific to *cbb*_3_-Cox and is not involved in the biogenesis of *aa*_3_-Cox. Therefore, we concluded that the Cu atoms needed for the formation of the heme-Cu_B_ binuclear centers in these two similar enzymes must be provided by distinct Cu uptake pathways.

## RESULTS

### Distribution of CcoA homologues in alphaproteobacteria.

**FIG 1:**
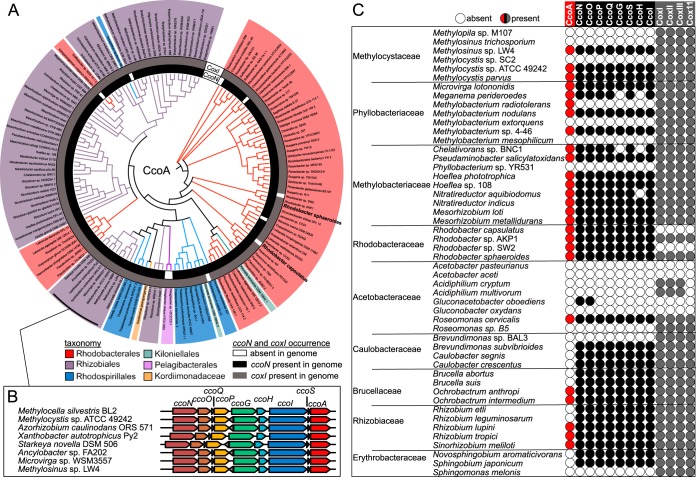
Presence of CcoA homologues encoded in alphaproteobacterial genomes. (A) Evolutionary relationships between the CcoA homologues identified in sequenced alphaproteobacterial genomes. Branch lengths were ignored, and branch points with Shimodaira-Hasegawa scores of <0.5 were deleted. Branches and nodes are colored by order as shown at the bottom of panel A. The presence of *ccoN* (inner circle) or *cox1* (outer circle) in each genome is represented by black or gray shading, respectively. Corresponding protein IDs are listed in Table S1. (B) Schematic representation of the *cbb*_3_-Cox structural (*ccoNOQP*) and assembly (*ccoGHIS*) gene clusters together with the *ccoA* homologue in the *Rhizobiales* genomes indicated. (C) Co-occurrence plot with circles indicating the presence or absence of *ccoA* (red or white, respectively), the *cbb*_3_-Cox structural and assembly gene clusters (black or white, respectively), and the *aa*_3_-Cox-related genes (gray or white, respectively). Not all species are shown because of space limitations, but for a complete profile and a summary, see Tables S1 and S2, respectively.

Using CcoA from *R. capsulatus* as a query, we identified 144 CcoA-like MFS proteins in 125 of the 327 alphaproteobacterial species interrogated, with several genomes containing up to three distinct copies of *ccoA*. We also compiled a phylogenetic profile of *ccoA* together with the presence of the *cbb*_3_-Cox and *aa*_3_-Cox structural genes (Fig. 1A). Most of the CcoA homologues were predicted to contain 12 transmembrane helices with conservation of the motifs MXXXM in helix 7 and HXXXM in helix 8, both of which are required for Cu uptake and for *cbb*_3_-Cox activity in *R. capsulatus* (37), with the exception of a group of CcoA-like proteins in *Rhizobiales* (Fig. 1B). These putative transporters also contained the two conserved motifs in helices 7 and 8 but were truncated at the C terminus, lacking predicted helix 12. Intriguingly, the genes encoding these truncated CcoA homologues were found right downstream of the *ccoNOQP* and *ccoGHIS* clusters, encoding the structural and assembly genes of *cbb*_3_-Cox, respectively (Fig. 1B), suggesting that the *Rhizobiales* CcoA-like proteins might play a role similar to that of *R. capsulatus* CcoA. For the complete set of data pertinent to Fig. 1, see Table S1 in the supplemental material.

10.1128/mBio.00065-18.1 TABLE S1  Co-occurrence of CcoA, *aa*_3_-Cox, and *cbb*_3_-Cox in alphaproteobacterial species. Download TABLE S1, XLSX file, 0.2 MB. Copyright © 2018 Khalfaoui-Hassani et al. 2018 Khalfaoui-Hassani et al. This content is distributed under the terms of the Creative Commons Attribution 4.0 International license.

**FIG 2:**
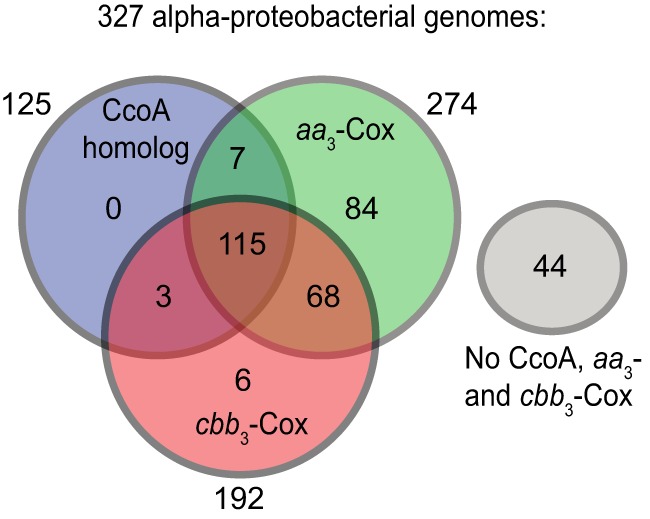
Distribution of CcoA, *aa*_3_-Cox, and *cbb*_3_-Cox homologues among the alphaproteobacterial genomes analyzed. Note that 44 genomes have no CcoA, *aa*_3_-Cox, or *cbb*_3_-Cox homologues, whereas 115 have all of them. Of the genomes that have no CcoA, 68 have both *aa*_3_-Cox or *cbb*_3_-Cox, while 84 have only *aa*_3_-Cox and 6 have only *cbb*_3_-Cox. Note that of the CcoA-containing genomes, only three have only *cbb*_3_-Cox and seven have only *aa*_3_-Cox.

We found that of the 327 alphaproteobacterial genomes analyzed, only 44 had no CcoA or Cox enzyme (Fig. 2; Table S2). Among the remaining genomes, 118 (62%) of 192 coding for *cbb*_3_-Cox (CcoNOQP) and 122 (46%) of 274 coding for *aa*_3_-Cox (CoxI, CoxII, and CoxIII) also contained CcoA. In contrast, 74 (23%) of these genomes had *cbb*_3_-Cox but not CcoA (mainly from *Caulobacterales*, *Brucella*, *Rhizobiaceae*, *Hyphomonadaceae*, *Rhodospirillaceae*, and *Sphingomonadales*), while 152 (46%) had *aa*_3_-Cox without CcoA (Fig. 2; Table S2). Thus, the data suggested a higher degree of co-occurrence of *ccoA* and *cbb*_3_-Cox than of *ccoA* and *aa*_3_-Cox. This co-occurrence was particularly evident in *Methylocystaceae* and *Methylobacteriaceae*, where species of the same genus would have both CcoA and *cbb*_3_-Cox or neither (Fig. 1C). In addition, we also observed strain differences; e.g., both *Paracoccus denitrificans* strains SD1 and PD1222 had *cbb*_3_-Cox and *aa*_3_-Cox but only strain PD1222 contained CcoA. Similarly, all of the *Rhizobium leguminosarum* strains analyzed had *cbb*_3_-Cox and *aa*_3_-Cox but individual biovars differed in the presence of CcoA. Finally, we found six species containing *cbb*_3_-Cox without CcoA and seven species containing CcoA but not *cbb*_3_-Cox, suggesting that CcoA-independent provision of Cu to *cbb*_3_-Cox and an additional unknown function(s) of CcoA, that is unrelated to Cu provision to the Cu_B_ center of this enzyme might exist in some species (Table S2).

10.1128/mBio.00065-18.2 TABLE S2  Distribution of CcoA, *cbb*_3_-Cox, and *aa*_3_-Cox among the alphaproteobacterial genomes analyzed in this study. Download TABLE S2, DOCX file, 0.01 MB. Copyright © 2018 Khalfaoui-Hassani et al. 2018 Khalfaoui-Hassani et al. This content is distributed under the terms of the Creative Commons Attribution 4.0 International license.

### Phenotypes of *ccoA* mutants of *R. sphaeroides*.

**FIG 3:**
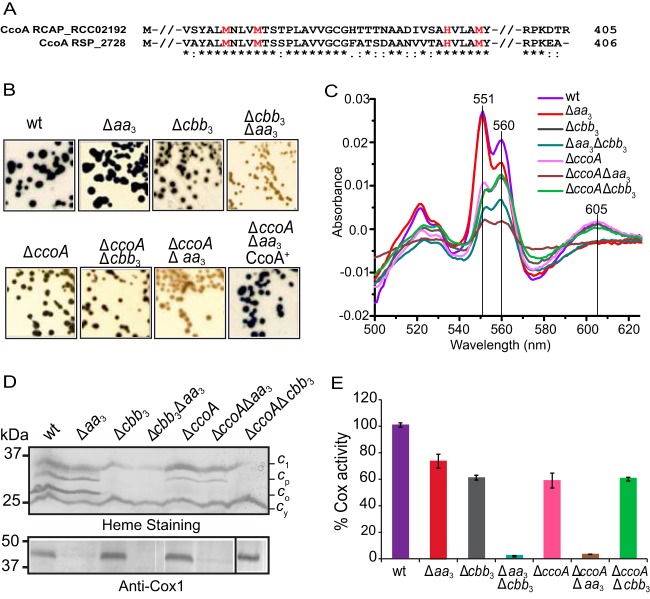
Met motifs of CcoA and phenotypic and functional characterization of *R. sphaeroides* Δ*ccoA* mutants. (A) Amino acid sequence alignment of the highly conserved region surrounding the Met and His motifs MXXXM and HXXXM (in red) from *R. capsulatus* CcoA (RCA_RCC02192) and its *R. sphaeroides* homologue (RSP_2726) (65% identity; 80% similarity). (B) Growth and NADI phenotypes of colonies of *R. sphaeroides* wild-type (wt) Ga and Δ*aa*_3_ (JS100), Δ*cbb*_3_ (MT001), and Δ*aa*_3_ Δ*cbb*_3_ (ME127) Cox mutants together with those of Δ*ccoA* (HW3), Δ*ccoA* Δ*cbb*_3_ (HW24), and Δ*ccoA* Δ*aa*_3_ (HW2) CcoA mutants. Cells were grown aerobically at 35°C on LB medium, and the presence of Cox activity was visualized by NADI staining (see Materials and Methods). Colonies that contain wild-type levels of Cox activity turn dark blue within a few seconds (NADI^+^), while those that have low or no Cox activity show lighter blue (NADI^slow^) or no blue staining (NADI^−^) upon longer exposure, respectively. Note that the Δ*ccoA* Δ*aa*_3_ mutant is NADI^–^ like the Δ*aa*_3_ Δ*cbb*_3_ mutant, unless it is complemented with a plasmid carrying a wild-type allele of *ccoA* (Δ*ccoA* Δ*aa*_3_ CcoA^+^). (C) Absorption difference spectra of membrane fractions of *R. sphaeroides* mutants recorded between 500 and 625 nm by using oxidized membrane preparations as the baseline and reducing the sample with an excess of sodium dithionite. The intensity of the peaks centered at 551, 560, and 605 nm indicates the contents of *c*-, *b*- and *a*-type hemes, respectively. (D) Steady-state levels of structural subunits of *cbb*_3_- and *aa*_3_-Cox enzymes in the membranes of *R. sphaeroides* mutants. (Top) Membrane preparations of *R. sphaeroides* mutants separated by SDS-PAGE and then stained with TMBZ. Four different cyts *c* (cyt *c*_1_ of the cyt *bc*_1_ complex, cyt *c*_p_ [CcoP] and *c*_o_ [CcoO] subunits of *cbb*_3_-Cox, and the membrane-attached electron carrier cyt *c*_y_) can be seen in the wild-type strain (39). In the Δ*ccoA* mutant, the steady-state levels of cyt *c*_p_ and especially cyt *c*_o_ are very low. (Bottom) Membrane preparations of *R. sphaeroides* strains resolved by SDS-PAGE and subjected to immunoblot analysis. The presence of the Cox1 subunit of *R. sphaeroides aa*_3_-Cox was identified with *P. denitrificans* Cox1 polyclonal antibodies that cross-react with it. The white lines seen on the blot next to some lanes are scanning artifacts and do not reflect spliced gels. (E) cyt *c* activity of membrane fractions of *R. sphaeroides* Δ*ccoA* mutants. Total Cox (*cbb*_3_-Cox plus *aa*_3_-Cox) activities were determined using membrane preparations of various *R. sphaeroides* strains by monitoring the rate of oxidation of reduced horse heart cyt *c*. *R. sphaeroides* wild-type strain Ga exhibited an activity of ~1.33 µmol of cyt *c* oxidized/min/mg of total membrane proteins, which was referred to as 100%. Three independent assays were carried out for each strain. The Δ*ccoA* Δ*aa*_3_ mutant has no activity, like the Δ*aa*_3_ Δ*cbb*_3_ mutant that lacks both Cox enzymes.

To understand the role of CcoA in the biogenesis of the Cu_B_ site of HCO and to test whether CcoA is involved in the provision of Cu to *aa*_3_-Cox, as it is in the provision of Cu to *cbb*_3_-Cox, we investigated the function of a CcoA ortholog in an organism that contains multiple HCOs. *R. sphaeroides* has a CcoA homologue (RSP_2726, previously annotated as a multidrug/metabolite efflux pump) containing the conserved Met motifs in transmembrane helices 7 and 8 (Fig. 3A). Unlike *R. capsulatus*, which is rare among the alphaproteobacterial species in having only one HCO (*cbb*_3_-Cox), *R. sphaeroides* also contains the canonical type A *aa*_3_-Cox. To assess the effect of lacking CcoA on both *cbb*_3_- and *aa*_3_-Cox activities, a *ccoA* deletion allele was introduced into appropriate *R. sphaeroides* strains. The wild-type (Ga) strain and the Δ*aa*_3_ (JS100 [38]) and Δ*cbb*_3_ (MT001 [39, 40]) mutant strains yielded the Δ*ccoA* (HW3) single mutant and the Δ*ccoA* Δ*aa*_3_ (HW2) and Δ*ccoA* Δ*cbb*_3_ (HW4) double mutants, respectively (Table S3). The Δ*cbb*_3_ Δ*aa*_3_ double mutant (ME127 [40]), lacking both *cbb*_3_- and *aa*_3_-Cox activities, served as a negative control (Table S3). The Cox activities of these strains were visualized qualitatively by α-naphthol and *N*′*N*′-dimethyl-*p*-phenylenediamine (NADI; blue) staining of colonies grown aerobically on enriched medium (Fig. 3B). The wild-type and Δ*aa*_3_ mutant strains were NADI^+^ (i.e., stained dark blue in seconds), whereas the Δ*cbb*_3_ mutant was NADI^slow^ (i.e., stained blue in a few minutes), indicating that *cbb*_3_-Cox provides most of the Cox activity under these growth conditions. The Δ*aa*_3_ Δ*cbb*_3_ double mutant was NADI^−^ (i.e., no blue staining after 15 min), consistent with the absence of both Cox enzymes (39). Both the Δ*ccoA* single mutant and the Δ*ccoA* Δ*cbb*_3_ double mutant had a NADI^slow^ phenotype, similar to that observed when only *aa*_3_-Cox activity (Δ*cbb*_3_) was present (Fig. 3B). Importantly, the double mutant lacking both CcoA and *aa*_3_-Cox (Δ*ccoA* Δ*aa*_3_) but containing the intact structural genes of *cbb*_3_-Cox was NADI^−^ like the double mutant (Δ*cbb*_3_ Δ*aa*_3_) lacking both Cox activities (Fig. 3B). Upon complementation with a plasmid carrying a wild-type allele of *R. sphaeroides ccoA*, both the single (Δ*ccoA*) and double (Δ*ccoA* Δ*aa*_3_) mutants lacking CcoA became NADI^+^ (Fig. 3B). Thus, the data indicated that in *R. sphaeroides*, the absence of *ccoA* affected *cbb*_3_-Cox, but not *aa*_3_-Cox, activity.

10.1128/mBio.00065-18.3 TABLE S3  Strains and plasmids used in this study. Download TABLE S3, DOCX file, 0.03 MB. Copyright © 2018 Khalfaoui-Hassani et al. 2018 Khalfaoui-Hassani et al. This content is distributed under the terms of the Creative Commons Attribution 4.0 International license.

The NADI^–^ phenotypes of *R. sphaeroides* Δ*ccoA* mutants were restored upon the addition of 5 μM Cu^2+^ to the growth medium, similar to that seen in the *R. capsulatus* Δ*ccoA* mutant (34). In contrast, the Δ*ccoA* Δ*cbb*_3_ double mutant, which has only a functional *aa*_3_-Cox, remained NADI^slow^ upon Cu^2+^ supplementation, suggesting that Cu^2+^ addition had no effect on *aa*_3_-Cox activity (data not shown).

### Absence of CcoA affects heme and subunit compositions of *cbb*_3_-Cox but not *aa*_3_-Cox.

To assess how the absence of CcoA affects *R. sphaeroides* HCO biogenesis, the *c*-, *b*-, and *a*-type heme contents of membrane fractions derived from appropriate mutants were analyzed by using optical difference (dithionite-reduced minus ferricyanide-oxidized) spectra. In membranes of a wild-type *R. sphaeroides* strain, prominent peaks around 605, 560, and 551 nm, corresponding to the *a*-, *b*-, and *c*-type hemes, respectively, were readily detectable (39) (Fig. 3C). As expected, a significant decrease in the 605-nm peak and in the 560- and 551-nm peaks was observed in the Δ*aa*_3_ and Δ*cbb*_3_ mutants, respectively (41). Note that in *R. sphaeroides* membranes, only *aa*_3_-Cox has *a*-type heme but other proteins besides *cbb*_3_-Cox contain *b*- and *c*-type hemes (e.g., cyt *bc*_1_) under the growth conditions tested. Accordingly, in the double mutant (Δ*cbb*_3_ Δ*aa*_3_) lacking both Cox enzymes, all three peaks decreased substantially compared with the wild-type strain (Fig. 3C), as reported earlier (39). Remarkably, in the Δ*ccoA* single mutant only the content of *b*- and *c*-type hemes decreased, as in the mutant lacking only *cbb*_3_-Cox (Δ*cbb*_3_) or the Δ*ccoA* Δ*cbb*_3_ double mutant. Moreover, in the Δ*ccoA* Δ*aa*_3_ double mutant, all three peaks, corresponding to the *a*-, *b*-, and *c*-type hemes, decreased drastically, similar to what was seen in the double mutant (Δ*cbb*_3_ Δ*aa*_3_) (Fig. 3C). In summary, the data showed that in the absence of CcoA, the content of *b*- and *c*-type hemes in the membrane fraction (corresponding partly to *cbb*_3_-Cox) decreased significantly, whereas the *a*-type heme content (corresponding to *aa*_3_-Cox) remained unchanged, consistent with CcoA being involved in *cbb*_3_-Cox, but not *aa*_3_-Cox, production. We emphasize that these data are merely semiquantitative because of the presence of other *b*- and *c*-type cyts (in addition to *cbb*_3_-Cox) whose content may vary in the presence or absence of different HCOs.

Next, the steady-state amounts of *cbb*_3_-Cox subunits present in membranes from appropriate mutants were examined by SDS-PAGE and 3,3′,5,5′-tetramethylbenzidine (TMBZ) staining, which specifically reveals membrane-bound *c*-type cyts (42). In wild-type *R. sphaeroides* membranes, four distinct *c*-type cyts, including the CcoO (cyt *c*_o_) and CcoP (cyt *c*_p_) subunits of *cbb*_3_-Cox, can be detected (Fig. 3D, top). As expected, in the absence of *aa*_3_-Cox, the *c*-type cyt profile remained unchanged, but in mutants lacking *cbb*_3_-Cox (Δ*cbb*_3_ and Δ*cbb*_3_ Δ*aa*_3_), cyt *c*_o_ and cyt *c*_p_ were not present, leaving only the cyt *c*_1_ subunit of cyt *bc*_1_ and the membrane-anchored electron carrier cyt *c*_y_. Remarkably, in strains lacking CcoA, such as the Δ*ccoA* and Δ*ccoA* Δ*aa*_3_ mutants, the amounts of cyt *c*_o_ and cyt *c*_p_ decreased at different levels, even though these strains contained an intact copy of the *cbb*_3_-Cox structural genes. These data, together with the spectral data showing that the amount of *b*-type heme, and hence that of CcoN, also decreased, indicated that production of *cbb*_3_-Cox was defective in the absence of CcoA. Finally, the presence of the Cox1 subunit of *aa*_3_-Cox was monitored by using polyclonal antibodies raised against Cox1 of *Paraccocus denitrificans aa*_3_-Cox (43) (Fig. 3D, bottom). As expected, Cox1 was absent from mutants lacking *aa*_3_-Cox, like the Δ*aa*_3_, Δ*cbb*_3_ Δ*aa*_3_, and Δ*ccoA* Δ*aa*_3_ mutant strains. However, it was readily detected in strains lacking CcoA (Δ*ccoA* mutant), *cbb*_3_-Cox (Δ*cbb*_3_ mutant), or both proteins (Δ*ccoA* Δ*cbb*_3_ mutant) at levels comparable to those of the wild type, in agreement with the Cu-containing Cox1 subunit of *aa*_3_-Cox being unaffected by the absence of CcoA in *R. sphaeroides*.

### Cox activities of mutants lacking CcoA.

The total cyt *c* oxidation activity (accounting for both *aa*_3_-Cox and *cbb*_3_-Cox activities) present in membranes of different *R. sphaeroides* strains was measured by using reduced horse heart cyt *c*. *R. sphaeroides* wild-type strain Ga exhibited an activity level of 1.33 μmol of cyt *c* oxidized/min/mg of total membrane proteins (referred to as 100%) (Fig. 3E). Addition of 200 μM KCN, a specific inhibitor of the HCO catalytic binuclear center, abolished this activity almost completely (96% inhibition). The mutants lacking *aa*_3_-Cox (Δ*aa*_3_ mutant) and *cbb*_3_-Cox (Δ*cbb*_3_ mutant) showed Cox activities corresponding to 73 and 62% of the wild-type level, respectively, whereas the Δ*cbb*_3_ Δ*aa*_3_ double mutant had no activity. A strain lacking only CcoA (Δ*ccoA* mutant) or both CcoA and *cbb*_3_-Cox (Δ*ccoA* Δ*cbb*_3_ mutant) showed similar amounts of Cox activity, 59 and 60% of the wild-type level, respectively. In contrast, a strain lacking both CcoA and *aa*_3_-Cox (Δ*ccoA* Δ*aa*_3_ mutant), although it contained intact *cbb*_3_-Cox structural genes, had no Cox activity, similar to a Δ*cbb*_3_ Δ*aa*_3_ double mutant (Fig. 3E). Therefore, the absence of CcoA affected only *cbb*_3_-Cox, and not *aa*_3_-Cox, in *R. sphaeroides*.

### Suppressors of Δ*ccoA* restore *cbb*_3_-Cox activity at the expense of Cu^2+^ hypersensitivity.

**FIG 4:**
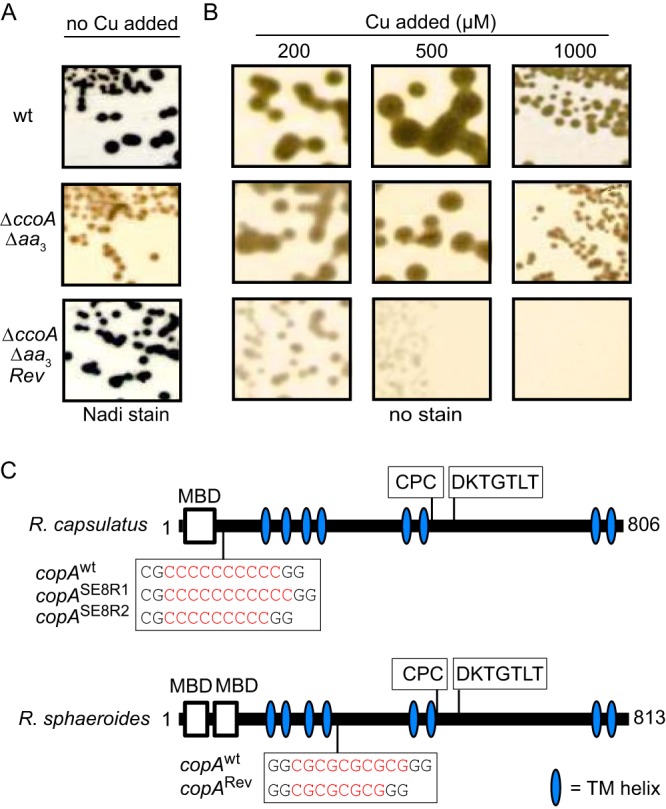
Bypass suppressors of *R. sphaeroides* mutants lacking CcoA are located in CopA and regain *cbb*_3_-Cox activity at the expense of Cu^2+^ hypersensitivity. (A) Spontaneous NADI^+^ bypass suppressors Δ*ccoA* Δ*aa*_3_/*Rev*_i_ that regained *cbb*_*3*_-Cox activity were isolated from the Δ*ccoA* Δ*aa*_3_ mutant. (B) The Δ*ccoA* Δ*aa*_3_/*Rev*_i_ suppressors exhibit hypersensitivity to Cu^2+^ compared with the wild type (wt) and the Δ*ccoA* Δ*aa*_3_ mutant. (C) The suppressor mutations corresponded to 2-bp (CG) deletions in *copA* (RSP_2829) of *R. sphaeroides* in a stretch of five CG repeats located immediately after the fourth transmembrane segment. They are compared with similar CcoA suppressors (CopA^SE8R1^ and CopA^SE8R2^) isolated previously from *R. capsulatus* (32). The latter mutations corresponded to a single-base-pair (C) indel located in a stretch of 10 C repeats of *R. capsulatus* CopA before its first transmembrane helix. In both species, the suppressor mutations led to translational frameshifts that abolished CopA activity, leaving intact the possibility of producing N-terminally truncated polypeptides that still carry the MBDs. The N-terminal MBD, the phosphorylation domain (DKTGT), and the transmembrane metal binding motif (CPC) are represented in CopA.

During the phenotypic characterization of Δ*ccoA* mutants, we observed that the NADI^–^ double mutant lacking both CcoA and *aa*_3_-Cox (Δ*ccoA* Δ*aa*_3_ mutant) readily yielded wild-type-like NADI^+^ revertants (Fig. 4A). Similar revertants had previously been obtained with *R. capsulatus* Δ*ccoA* mutants, and their characterization showed that these suppressor mutations restored *cbb*_3_-Cox deficiency and conferred Cu^2+^ sensitivity (32). Using whole-genome sequencing, we determined that these mutations were single base-pair indels in a rare stretch of 10 conserved cytosine base pairs located in *copA*, which encoded the P_1B_-type ATP-dependent Cu exporter (CopA) (32). These indels caused translational frameshifts that inactivated *copA* and increased cellular Cu content and Cu^2+^ sensitivity (32).

Intrigued by the occurrence of similar revertants of *R. sphaeroides*, we retained four independent NADI^+^ derivatives (HW2R_1_ to HW2R_4_) of the Δ*ccoA* Δ*aa*_3_ double mutant (Table S3) and tested their Cu^2+^ tolerance in enriched medium. Indeed, they were hypersensitive to Cu^2+^ (above ~200 μM) compared with their wild-type and Δ*ccoA* Δ*aa*_3_ mutant parents (tolerant to ~1 mM) (Fig. 4B). Thus, similar to *R. capsulatus*, these *R. sphaeroides* revertants regained *cbb*_3_-Cox activity at the expense of becoming hypersensitive to Cu^2+^. DNA sequencing of the genomic copies of *R. sphaeroides copA* (RSP_2890) (44) from these revertants showed that they all contained two base-pair (CG) deletions in *copA* (Fig. 4C). Remarkably, these deletions were located in a region of *copA* containing five consecutive CG repeats, presumably causing translational frameshifts that inactivated *copA* and increased the Cu^2+^ sensitivity of cells. The data indicated that in *R. sphaeroides*, as in *R. capsulatus*, suppression of the CcoA defect occurred via mutations (two base-pair CG deletions and single base-pair C indels, respectively), inducing translational frameshifts that inactivated CopA.

### Distribution of the CcoA family among *Bacteria* and *Eukarya*.

**FIG 5:**
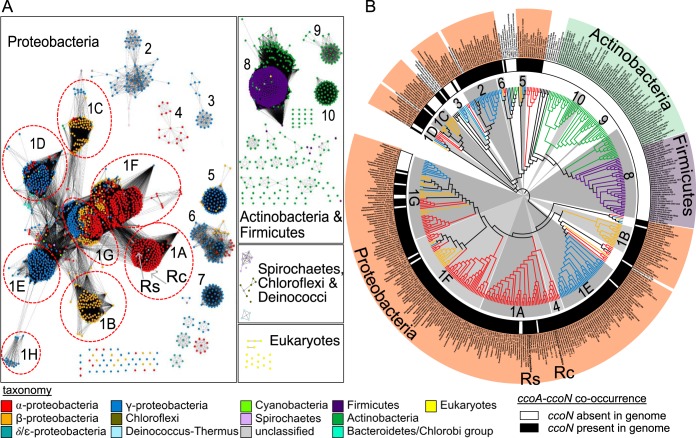
The CcoA-like family of putative transporters. (A) Protein similarity network of CcoA-like putative transporters identified in the Uniprot database. Each node (circle) represents a single protein sequence, and each edge (solid line) represents similarity between two proteins (threshold set at an alignment score of 80). Nodes are colored by taxonomy as shown at the bottom, and cluster designations (1 to 10) correspond to Table S4 and Fig. S1 PSN_CcoA.cys, which can be viewed in detail by using Cytoscape software. The locations of the nodes representing CcoA from *R. sphaeroides* (Rs) and *R. capsulatus* (Rc) are shown with gray arrows. (B) Evolutionary relationship between CcoA homologues identified in the SEED database. Branch lengths were ignored, and branch points with Shimodaira-Hasegawa scores of <0.5 were deleted. Branches are colored by taxonomy as shown at the bottom. The presence of *ccoN* (inner circle) in each genome is represented by black shading. Gray shading is used to color those clades that correspond to the numbered clusters (1A to 10) shown in panel A. Corresponding protein IDs are listed in Table S5. The positions of CcoA of *R. sphaeroides* (Rs) and *R. capsulatus* (Rc) in the tree are also indicated. Note that *ccoA* is present in *Actinobacteria* and *Firmicutes* (clusters 8 to 10) that are devoid of *ccoN* (i.e., *cbb*_3_-Cox).

Given the functional specificity of CcoA for *cbb*_3_-Cox in *Rhodobacter* species, we widened our bioinformatic search for CcoA-like MFS transporters beyond the alphaproteobacteria and queried their co-occurrence with CcoN in the SEED database (45). We found CcoA homologues in all major classes of *Proteobacteria*, *Bacteroidete*s, and *Spirochaetia* and in all major divisions of the *Terrabacteria* group, including *Chloroflexi* and *Deinococcus* (Fig. 5A). Moreover, we found that most of the genomes that contained CcoA also encoded CcoN (i.e., *cbb*_3_-Cox), except *Actinobacteria* and *Firmicutes* (Fig. 5B). The CcoA-like proteins were also present in the nuclear genomes of eukaryotic algae, with both primary and secondary plastids, in two fungal genomes from *Chytridiomycota* (Fig. 5A), in addition to the group of *Actinobacteria* and *Firmicutes* (Fig. 5B), which are known to lack *cbb*_3_-Cox (26). Remarkably, these “orphan” CcoA-like transporters encountered in organisms lacking *cbb*_3_-Cox still contained the conserved MXXXM and HXXXM motifs in helices 7 and 8, suggesting that they might also transport Cu to other protein targets. Finally, we note that, similar to alphaproteobacterial genomes, about one-third of *ccoN*-containing organisms also contain *ccoA* and exhibited species level variation in its presence, which is particularly evident in *Vibrio* (Table S4). For the set of data pertinent to Fig. 5A and B, see Tables S4 and S5, respectively.

10.1128/mBio.00065-18.4 TABLE S4  Protein similarity network of CcoA-like putative transporters obtained by using the SEED database. Download TABLE S4, XLSX file, 0.8 MB. Copyright © 2018 Khalfaoui-Hassani et al. 2018 Khalfaoui-Hassani et al. This content is distributed under the terms of the Creative Commons Attribution 4.0 International license.

10.1128/mBio.00065-18.5 TABLE S5  Evolutionary relationship between CcoA homologues and the corresponding phylogenetic tree. Download TABLE S5, XLSX file, 0.2 MB. Copyright © 2018 Khalfaoui-Hassani et al. 2018 Khalfaoui-Hassani et al. This content is distributed under the terms of the Creative Commons Attribution 4.0 International license.

## DISCUSSION

The *R. capsulatus* MFS-type transporter CcoA is the prototypical bacterial Cu importer and the key Cu provider to *cbb*_3_-Cox under limited Cu availability (32, 34, 37). Earlier, we observed that *R. capsulatus* mutants lacking either *cbb*_3_-Cox (Δ*ccoNOQP*) or CcoA (Δ*ccoA*) contained similar smaller amounts of total cellular Cu (~80% of the wild-type amount) (46), suggesting that the Cu imported by CcoA is allocated primarily to *cbb*_3_-Cox biogenesis. To assess the functional specificity of CcoA toward other cuproenzymes, we initiated a broad-based comparative genomic study to examine the presence of CcoA homologues and their co-occurrence with cyt *c* oxidases in organisms of known genome sequences. We found that the CcoA-like transporters are widespread in bacteria and some microbial eukaryotes. They are present in all major classes of *Proteobacteria*, *Bacteroidetes*, *Spirochaetia*, and *Terrabacteria*, as well as in the nuclear genomes of eukaryotic algae and fungi (Table S1). Interestingly, our all-inclusive bioinformatic analyses showed that not all CcoA family members are involved in *cbb*_3_-Cox biogenesis. Numerous species that have no *cbb*_3_-Cox, such as *Actinobacteria* and *Firmicutes* species, still contained CcoA-like transporters that possibly perform other functions that have yet to be uncovered. A closer look to the group of alphaproteobacteria showed that about one-third of these species contained at least one CcoA homologue together with the genes encoding *cbb*_3_-Cox or *aa*_3_-Cox, showing a high degree of co-occurrence of HCO with CcoA. Finally, similar to some genera of alphaproteobacteria, we observed species level variations in the presence of *ccoA*, which were particularly evident in *Vibrio* (Table S4), which may reflect that CcoA provides a selective advantage in some environmental niches.

**FIG 6:**
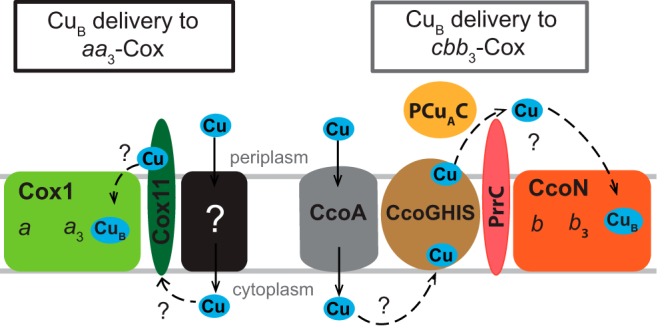
Schematic comparison of Cu_B_ incorporation into the active sites of bacterial *aa*_*3*_- and *cbb*_3_-Cox. A complete understanding of the pathways of Cu uptake and delivery to the heme-Cu_B_ binuclear site of HCOs is still missing. All known components involved in the biogenesis of the Cu_B_ center of bacterial *aa*_3_- and *cbb*_3_-Cox are depicted. In the case of *aa*_3_-Cox, the Cox1 subunit is thought to receive Cu_B_ from the Cu chaperone Cox11 either directly or via an unknown partner(s). How Cu is initially conveyed to Cox11 remains unknown. A putative Cu importer of unknown identity (black box) that is possibly functionally similar to mitochondrial Pic2 (71) is included. In the case of *cbb*_3_-Cox, Cu is imported by CcoA and is conveyed by the CcoGHIS, PCuAC, and PrrC (PccA and SenC homologs) assembly components to the Cu_B_ site of the CcoN subunit by a mechanism that remains elusive. Question marks indicate unknown Cu transfer steps.

Using *R. sphaeroides*, which contains both *cbb*_3_- and *aa*_3_-Cox (from C and A HCO families, respectively) and an ortholog of *R. capsulatus* CcoA (RSP_2726), we tested experimentally whether CcoA could also provide Cu to the canonical *aa*_3_-Cox, whose biogenesis has been studied (43, 47). Physiological, genetic, and biochemical data gathered by using appropriate Δ*ccoA* mutants lacking either *cbb*_3_- or *aa*_3_-Cox established unequivocally that the absence of CcoA affected *cbb*_3_-Cox, but not *aa*_3_-Cox, production in this organism. Earlier work had shown that the absence of the Cu chaperone Cox11, which is required for Cu_B_ insertion into *aa*_3_-Cox, had no effect on *cbb*_3_-Cox production in *R. sphaeroides* (25) or *Pseudomonas pseudoalcaligenes* KF707 (48). Therefore, we concluded that the Cu atoms inserted into the binuclear centers of the *cbb*_3_-Cox and *aa*_3_-Cox enzymes are not only delivered by distinct pathways but also provided by different uptake systems (Fig. 6).

CcoA being an exclusive Cu importer for *cbb*_3_-Cox was rather unexpected, especially because the catalytic subunits and the heme-Cu binuclear centers of all HCOs are very similar (11, 49). The existence of specialized Cu trafficking pathways for different cuproproteins has been documented in different organisms (50), and their specificity is generally conferred by target-specific chaperones rather than transporters (51). Thus, the independent Cu uptake systems operating during the biogenesis of different HCOs and the specificity of CcoA for *cbb*_3_-Cox are intriguing. Since the Sco-like and PCu_A_C-like Cu chaperones are involved in the biogenesis of both *cbb*_3_-Cox (23, 27, 33) and *aa*_3_-Cox (19, 22, 24, 52), they are less likely to confer specificity. Thus, a possibility is that CcoA may do so by conveying Cu either directly or via an unknown partner, to CcoI, which is the P_1B_-type ATPase required for *cbb*_3_-Cox production (29) (Fig. 6). Interestingly, the physical clustering of *ccoA* with the *cbb*_3_-Cox assembly genes *ccoGHIS* in members of the order *Rhizobiales* (Fig. 1B) suggests that these proteins function together and possibly interact during *cbb*_3_-Cox production. Undoubtedly, under low Cu availability, the occurrence of a membrane-integral complex containing both CcoI and CcoA would be advantageous for efficient biosynthesis of *cbb*_3_-Cox.

In both *R. sphaeroides* and *R. capsulatus* mutants lacking CcoA, the defect in *cbb*_3_-Cox production can be restored by providing a high concentration of exogenous Cu^2+^, which leads to an increase in cellular Cu content (34). The components of this putative CcoA-independent low-affinity Cu uptake pathway remain unknown. However, this pathway still relies on CcoI, whose absence cannot be palliated by Cu^2+^ supplementation, to provide Cu to *cbb*_3_-Cox. Alternatively, the defect in *cbb*_3_-Cox biogenesis can be bypassed via frameshift mutations in *copA*, which encodes the P_1B_-type ATPase involved in Cu export and detoxification, resulting in inactivation of CopA and consequent greater cellular Cu content and hypersensitivity to Cu^2+^ (32). Elucidation of how the Cu imported by CcoA is conveyed to CcoI is needed to understand how the increase in cellular Cu content bypasses the role of CcoA in *cbb*_3_-Cox biogenesis.

The molecular natures and locations of the suppressor mutations that inactivate CopA are different between the two *Rhodobacter* species. In *R. capsulatus*, these mutations are single base-pair C indels in a region of *copA* where 10 consecutive C base-pairs are located (bp 230 to 239) (32), whereas in *R. sphaeroides*, they are two base-pair CG deletions in a region of *copA* where five consecutive CG repeats are present (bp 863 to 872). Hypermutable nucleotide tandem repeats (NTRs), which are prone to DNA slippage during replication and increased recombination, are widespread in genomes of different organisms (53, 54), and they can reversibly inactivate or regulate the expression of specific coding sequences (55). Computational analyses suggested that in prokaryotes, the monomeric NTRs of G/C (e.g., C repeats of *R. capsulatus copA*) are more mutagenic than dimeric (e.g., CG repeats of *R. sphaeroides copA*) or trimeric NTRs (56). The different types of mutagenic NTRs located in *copA* may reflect different strategies used for Cu homeostasis governing Cu availability to *cbb*_3_-Cox via CcoA-independent pathways.

P_1B_-type ATPases such as CopA and CcoI contain conserved domains for ATP binding and for phosphorylation, in addition to their N-terminal metal-binding domains (MBDs), harboring a Cu-binding CXXC motif and a membrane-embedded Cu binding site (CPX) (57) (Fig. 4C). The frameshift mutations that inactivate CopA still conserve the genetic ability to produce truncated N-terminal CopA derivatives with intact N-terminal MBDs that, if produced and stable, could hypothetically facilitate Cu delivery to *cbb*_3_-Cox. An *R. capsulatus* CopA derivative would become soluble with a single MBD, whereas an *R. sphaeroides* CopA derivative would remain membrane attached and conserve its two MBDs, reminiscent of the Cu chaperone CupA in *Streptococcus pneumoniae*. The membrane-anchored CupA protein enhances Cu sequestration and mediates its binding to the MBD of CopA as an adaptation to Cu toxicity (58). *Arabidopsis thaliana* chaperone PCH1 is produced by alternative splicing of the P_1B_-type Cu^+^ ATPase PAA1 pre-mRNA and acts as its specific Cu chaperone (59). In *Escherichia coli*, a fragment of CopA containing the N-terminal MBD, resulting from programed ribosomal frameshifting during the translation of *copA* mRNA, is able to bind Cu and increase tolerance of Cu toxicity (60, 61). The molecular mechanisms underlying these cases are distinct from the NTR mutations in *copA*, yet they reflect similar responses that organisms have evolved to maintain Cu homeostasis and avoid its toxicity.

The isolation of mutations in *copA* of both *R. capsulatus* and *R. sphaeroides* may suggest that CcoA is not required for *cbb*_3_-Cox metalation, depending on the mechanisms of Cu homeostasis used by the organisms. Indeed, our comparative genomic analyses indicated that CcoA-like MFS proteins are absent from about one-third of *cbb*_3_-Cox-containing alphaproteobacterial species. That *cbb*_3_-Cox metallation in these species does not require CcoA while it does so in *R. capsulatus* and *R. sphaeroides* under low Cu availability is intriguing. These species may have other Cu acquisition pathways, similar to *R. capsulatus ccoA* mutants at high Cu^2+^ concentrations. As an example, *P. denitrificans* PD1222 has an MFS-type CcoA Cu importer and a typical P_1B_-type ATPase CopA ortholog with an N-terminal heavy-metal-associated (HMA) domain acting as its MBD. In contrast, *P. denitrificans* SD1 does not have CcoA but has a CopA homologue with a different MBD, an N-terminal TRASH domain (62). These differences are in agreement with the proposal that the *copA* NTR mutations occurring after the N-terminal MBD of CopA in both *Rhodobacter* species may result in HMA-containing derivatives acting as chaperones. Further investigation of these species and characterization of different strains with respect to their CcoA-independent Cu trafficking pathways will be informative.

In summary, this work established that Cu incorporation into the catalytic site of different HCOs, in particular *cbb*_3_-Cox and *aa*_3_-Cox, occurs not only via distinct delivery pathways but also via distinct uptake pathways (Fig. 6). While the MFS-type transporter CcoA is required for Cu incorporation into *cbb*_3_-Cox, it is not involved in the metallation of *aa*_3_-Cox. The occurrence of dedicated Cu uptake pathways, critical for the maintenance of intracellular Cu homeostasis, might be an evolutionary example of different strategies to improve fitness encountered in many organisms.

## MATERIALS AND METHODS

### Bacterial strains, plasmids, and growth conditions.

The *E. coli* and *R. sphaeroides* strains and plasmids used in this study are listed in Table S3. The standard molecular biology techniques used are described in reference 63, and all plasmid and strain constructions are described below Table S3. *E. coli* strains were grown at 37°C in Luria-Bertani (LB) broth supplemented with 100, 50, 50, 12.5, and 12 μg/ml (final concentrations) ampicillin, kanamycin (Kan), spectinomycin (Spe), tetracycline (Tet), and gentamicin (Gen), respectively (46). *R. sphaeroides* strains were grown in either minimal (64) or LB medium supplemented with 10, 10, 2.5, and 1 μg/ml (final concentrations) Kan, Spe, Tet, and Gen, respectively (39).

### Biochemical and spectroscopic techniques.

*R. sphaeroides* cells grown under semiaerobic conditions on LB medium were harvested and resuspended in 50 mM Tris-HCl (pH 7.2), 1 mM KCl assay buffer. Intracytoplasmic membrane vesicles (chromatophores) were prepared as previously described (65). The protein concentration of membrane fractions was determined with the bicinchoninic acid assay (Sigma, Inc.). Visualization of *c*-type cyts was done by TMBZ staining following the separation of ~200 μg of total membrane proteins by 15% SDS-PAGE as done earlier (42). Immunoblot analysis to identify *R. sphaeroides* Cox1 was done with ~40 μg of total membrane proteins separated by 12% SDS-PAGE. Proteins were transferred onto polyvinylidene difluoride membranes and incubated with *P. denitrificans* anti-Cox1 specific polyclonal antibodies cross-reacting with *R. sphaeroides* protein (47). Alkaline phosphatase-conjugated secondary antibodies and 5-bromo-4-chloro-3-indolyl phosphate (BCIP)–nitroblue tetrazolium were used for visualization of Cox1 polypeptide.

Visible spectra were taken with 50 μg of total membrane proteins in 1 ml of assay buffer containing 0.2% *n*-dodecyl-β-d-maltoside (DDM). Samples were oxidized by the addition of a few grains of potassium ferricyanide, and the absorption spectra taken between 480 and 660 nm were saved as a baseline. After reduction of the samples by the addition of a small amount of sodium dithionite, the spectra were rerecorded in the same wavelength range (39).

### Determination of Cox activities.

The *cbb*_3_-Cox activity of colonies was visualized by using the NADI reaction (α-naphthol + *N*′*N*′-dimethyl-*p*-phenylenediamine → indophenol blue + H_2_O) by staining the plates with a 1:1 (vol/vol) mixture of 35 mM α-naphthol and 30 mM *N*′,*N*′-dimethyl-*p*-phenylenediamine (66). Colonies with *cbb*_3_-Cox activity exhibited dark blue staining (NADI^+^) within 30 s to 1 min, while those with low activity or lacking it showed light blue (NADI^slow^) or no staining (NADI^−^) up to 15 min, respectively. Total *aa*_3_-Cox and *cbb*_3_-Cox activity levels were determined with reduced cyt *c* as a substrate as done previously (39). Chromatophore membranes were solubilized at room temperature by the addition of 1 mg of DDM/mg of total proteins. Activity assays were initiated by the addition of ~10 μg of solubilized membranes to 1 ml of assay buffer containing 25 μM reduced cyt *c*. Rates of cyt *c* oxidation were determined by monitoring the time-dependent decrease in absorbance at 550 nm and expressed in micromoles of cyt *c* oxidized/min/mg of total membrane proteins by using the extinction coefficient at 550 nm for cyt *c* (ε_550_ = 20.0 mM^−1^ cm^−1^). The specificity of Cox activity was confirmed by inhibition with 200 μM KCN, a specific inhibitor of HCO enzymes, which stopped cyt *c* oxidation almost completely. Any residual cyanide-insensitive cyt *c* oxidase activity (air oxidation was negligible) was subtracted from the final rates.

### Bioinformatic analysis.

Genes encoding CcoA-like, CcoN, and Cox1 proteins were identified in the SEED database (45). In addition to amino acid sequence similarity, annotation of a protein as being CcoA-like required conservation of the MXXXM and HXXXM motifs of transmembrane helices 7 and 8. Patterns of co-occurrence and genomic colocalization were detected with the set of tools for comparative genome analysis available in SEED. For the phylogenetic trees of CcoA-like proteins, full-length amino acid sequences (Tables S1, S4, and S5) were aligned through the CIPRES web portal (67) with MAFFT on XSEDE (v. 7.305) (68) and an approximate maximum-likelihood estimation was performed with FastTreeMP on XSEDE (v. 2.1.9) (69). The resulting phylogenetic trees were visualized and annotated with the Interactive Tree of Life (iTOL) tool (70). A comprehensive identification of CcoA homologues in sequenced genomes was performed with a protein similarity network as implemented with the EFI-EST tool (http://efi.igb.illinois.edu/efi-est/) with *R. capsulatus* CcoA as the seed sequence, an E value of 1E-4 for the blast search, and an alignment score of 80. EFI retrieved 2,490 proteins (see Table S4), which were incorporated into the network and visualized with the yFiles organic layout provided with the Cytoscape software (http://www.cytoscape.org).

10.1128/mBio.00065-18.6 TABLE S6  Primers used in this study. Download TABLE S6, DOCX file, 0.01 MB. Copyright © 2018 Khalfaoui-Hassani et al. 2018 Khalfaoui-Hassani et al. This content is distributed under the terms of the Creative Commons Attribution 4.0 International license.
