# Supplementary material for: Widespread Distribution and Functional Specificity of the Copper Importer CcoA: Distinct Cu Uptake Routes for Bacterial Cytochrome c Oxidases
Source: mBio. 2018 Feb 27;9(1):e00065-18. doi: 10.1128/mBio.00065-18 (PMC5829832; doi:10.1128/mBio.00065-18)
Supplement: TABLE S2 [file mbo001183741st2.docx]

**Table S2.** Distribution of CcoA, *cbb*_3_-Cox and *aa*_3_-Cox among the 327 alpha-proteobacterial genomes analyzed.

| **Protein(s)** | # of genomes encoding protein(s) | # of genomes relative to total # of genomes |
| --- | --- | --- |
| *aa*_3_-Cox | 274 | 84% |
| *cbb*_3_-Cox | 192 | 59% |
| CcoA homolog | 125 | 38% |
| CcoA and *cbb*_3_-Cox | 118 (61%)^[[1]](#footnote-1)^ | 36% |
| CcoA and *aa*_3_-Cox | 122 (45%)^[[2]](#footnote-2)^ | 37% |
| *aa*_3_-Cox and *cbb*_3_-Cox | 183 | 56% |
| CcoA, *aa*_3_-Cox, and *cbb*_3_-Cox | 115 (63%)^[[3]](#footnote-3)^ | 35% |
| *aa*_3_-Cox and no CcoA | 152 (55%)^1^ | 46% |
| *cbb*_3_-Cox and no CcoA | 74 (39%)^2^ | 23% |
| *aa*_3_-Cox, *cbb*_3_-Cox, no CcoA | 68 (37%)^3^ | 21% |
| CcoA, no *aa*_3_-Cox, no *cbb*_3_-Cox | 0 | 0% |
| CcoA and *cbb*_3_-Cox, no *aa*_3_-Cox | 3 | 0% |
| CcoA and *aa*_3_-Cox, no *cbb*_3_-Cox | 7 | 2% |
| No CcoA, no *aa*_3_-Cox, no *cbb*_3_-Cox | 44 | 13% |

^1^Portion of *cbb*_3_-Cox containing genomes

^2^Portion of *aa*_3_-Cox containing genomes

^3^Portion of *aa*_3_-Cox and *cbb*_3_-Cox containing genomes

1. [↑](#footnote-ref-1)
2. [↑](#footnote-ref-2)
3. [↑](#footnote-ref-3)
