# Supplementary material for: Widespread Distribution and Functional Specificity of the Copper Importer CcoA: Distinct Cu Uptake Routes for Bacterial Cytochrome c Oxidases
Source: mBio. 2018 Feb 27;9(1):e00065-18. doi: 10.1128/mBio.00065-18 (PMC5829832; doi:10.1128/mBio.00065-18)
Supplement: TABLE S3 [file mbo001183741st3.docx]

**Table S3.** Strains and plasmids used in this study.

|  | **Relevant properties** | **Reference** |
| --- | --- | --- |
| ***Strains****^a^* |  |  |
| *E. coli* |  |  |
| HB101 | F^-^ Δ(*gpt-proA*)62 *leuB6 supE44 ara*-14 *galK2 lacY* 1 Δ(*mcrC-mrr*) *rpsL20* (Str^R^) *xyl-5 mtl-1recA*13; Str^R^ | Promega |
| S17.1λpir | *recA* *thi* *pro* *hsd*R^-^M^+^ *RP4:2-Tc:Mu:kan* Tn7 λpir; Tp^R^ Str^R^ | ([1](#_ENREF_1)) |
| *R. sphaeroides* |  |  |
| Ga | Wild-type | ([2](#_ENREF_2)) |
| JS100 | Δ*ctaD::spe*; Spe^R^ | ([3](#_ENREF_3)) |
| MT001 | Δ*ccoNO::kan*; Kan^R^ | ([4](#_ENREF_4)) |
| ME127 | Δ*ctaD::spe* Δ*ccoNO::kan*; Spe^R^ Kan^R^ | ([4](#_ENREF_4)) |
| HW1 | Δ*ccoA::kan* derivative of Ga; Kan^R^ | This work |
| HW2 | Δ*ccoA::kan* Δ*ctaD::spe* derivative of JS100; Spe^R^ Kan^R^ | This work |
| HW3 | Δ*ccoA::spe* derivative of Ga; Spe^R^ | This work |
| HW4 | Δ*ccoNO::kan* Δ*ccoA::spe* derivative of MT001; Spe^R^ Kan^R^ | This work |
| HW2_i= 1-4_ | Nadi^+^ Cu^R^ revertants of HW2 (CG deletion in *copA*); Spe^R^ Kan^R^ | This work |
| ***Plasmids*** |  |  |
| pBluescriptII | pBS cloning vector; Amp^R^ | Stratagene |
| pRK2013 | Conjugation helper; Kan^R^ | ([1](#_ENREF_1)) |
| pRK415 | Broad host range vector; Tet^R^ | ([1](#_ENREF_1)) |
| pZJD29a | Non replicative *Rhodobacter* vector with R6K ori, *sacB*; Gen^R^ | ([5](#_ENREF_5)) |
| pSP1 | pBS derivative carrying 1.4 kb *BamHI-XbaI* fragment of *R. sphaeroides ccoA*; Amp^R^ | This work |
| pBK96 | pSP1 derivative carrying a 300 base pair deletion in *ccoA*; Amp^R^ | This work |
| pBK97 | pBK96 derivative carrying Δ*ccoA::kan*; Amp^R^ Kan^R^ | This work |
| pHW1 | pZJD29a derivative carrying Δ*ccoA::kan*; Gen^R^ Kan^R^ | This work |
| pHW2 | pBK96 derivative carrying Δ*ccoA::spe*; Amp^R^ Spe^R^ | This work |
| pHW3 | pZJD29a derivative carrying Δ*ccoA::spe*; Gen^R^ Spe^R^ | This work |
| pYZ31 | pBS derivative with 2.1 kb *XbaI-KpnI* fragment carrying entire *R. sphaeroides* *ccoA*; Amp^R^ | This work |
| pYZ32 | pRK415 derivative with *R. sphaeroides* *ccoA* on the *XbaI-KpnI* fragment of pYZ31; Tet^R^ | This work |
| pYZ33 | pBS derivative with 4.4 kb *XbaI-KpnI* fragment carrying *R. sphaeroides* *copA* region; Amp^R^ | This work |
| pYZ35 | pBS derivative with 4.4 kb *XbaI-KpnI* fragment carrying *copA* region from HWR2_i1_ (*copA*^Rev1^); Amp^R^ | This work |
| pYZ36 | pBS derivative with 4.4 kb *XbaI-KpnI* fragment carrying *copA* region from HWR2_i2_ (*copA*^Rev2^); Amp^R^ | This work |
| pYZ37 | pBS derivative with 4.4 kb *XbaI-KpnI* fragment carrying *copA* region from HWR2_i3_ (*copA*^Rev3^); Amp^R^ | This work |
| pYZ38 | pBS derivative with 4.4 kb *XbaI-KpnI* fragment carrying *copA* region from HWR2_i4_ (*copA*^Rev4^); Amp^R^ | This work |

^a^*R. sphaeroides* strains were derived from Sistrom’s Ga, which is a wild-type “green” derivative.

**Construction of various plasmids and strains listed in Table S3.**

A portion of *R. sphaeroides* *ccoA* homolog (RSP_2726) was amplified using the CcoAs-BamHIFor and XbaIRev1 primers and genomic DNA from strain Ga (**Table S6,** Supplemental Material), producing a 1.4 kb DNA fragment that was cloned into the corresponding restriction sites of plasmid pBlueScript II (pBSII) to yield pSP1. Using this plasmid as a template, the 3’ end of *ccoA* and the BamHI site were PCR amplified with CcoAs-StuIFor primer, overlapping a 900 base pair region of *ccoA*, and the commercial T3 primer. Similarly, using pSP1 as a template the 5’ end of *ccoA* and the XbaI site were PCR amplified with CcoAs-StuIRev primer, overlapping a 600 base pair region of *ccoA*, and the commercial T7 primer (**Table S6,** Supplemental Material). These fragments were digested with BamHI, XbaI and StuI, mixed together and ligated to pBSII digested with BamHI and XbaI yielding pBK96 (**Table S3**), which carried a ~300 base pair deletion terminating with the unique StuI restriction site in *ccoA*. A Kan^R^ gene cassette isolated from pMA117 by SalI digestion was treated with Klenow fragment and blunt-end ligated to pBK96 previously digested with StuI restriction enzyme to yield pBK97 with the *ccoA::kan* allele. Similarly, a Spe^R^ gene cassette isolated from pHP45Ω was inserted at the StuI site of pBK96 to yield pHW2 with the *ccoA::spe* allele. These mutant alleles were cloned using the BamHI and XbaI sites into the plasmid pZJD29a (5), which contains the *sacB* gene (lethal in the presence of sucrose) and lacks a functional origin of replication in *R. sphaeroides*, to yield pHW1 (*ccoA::kan*) and pHW3 (*ccoA::spe*) derivatives, maintained in an *E. coli* Pir^+^ background (**Table S3***,* Supplemental Material). A 2.1 kb DNA fragment carrying full length *R. sphaeroides* *ccoA* (Rsp_2726) was PCR amplified using genomic DNA from strain Ga with Rsp_2726ForX and Rsp_2726RevK primers (**Table S6,** Supplemental Material), digested with XbaI and KpnI enzymes and cloned into the same sites of pBSII to yield pYZ31. The pYZ31 clone was verified by DNA sequencing and digested with XbaI and KpnI, and the obtained fragment transferred to pRK415 using the same restriction sites to yield pYZ32 (**Table S3*,*** Supplemental Material). The 4.4 kb DNA fragment carrying a full-length *R. sphaeroides* *copA* (RSP_2890) was amplified by PCR using the primers CopAopForK and CopARevX (**Table S6,** Supplemental Material) and genomic DNA from strain Ga (**Table S3*,*** Supplemental Material), and cloned into pBSII plasmid after KpnI and XbaI digestions to yield pYZ33. Similarly, the plasmids pYZ35 to pYZ38 were obtained using genomic DNAs from HW2 revertants (HWR2_i=1 to 4_), respectively (**Table S3*,*** Supplemental Material). All *copA* clones were confirmed by DNA sequencing using primers CopAln1F, CopAln1R, CopAln2F and CopAln2R (**Table S6,** Supplemental Material). Plasmids pHW1 and pHW3 were mobilized from *E. coli* into appropriate *R. sphaeroides* strains via biparental crosses using the donor strain S17-1 as performed earlier (1, 5) (**Table S3*,*** Supplemental Material). The desired chromosomal knock-out mutants were isolated by selection for antibiotic and sucrose resistance, confirmed by PCR amplification and subsequent DNA sequencing of the corresponding chromosomal region. Triparental mating, using helper plasmid pRK2013, was used to complement the desired *R. sphaeroides* mutants with plasmid pRK415 derivatives carrying wild-type alleles of *R. sphaeroides* *ccoA* or *copA*, as done earlier (2, 83) (**Table S3*,*** Supplemental Material).

**Related References**

1. Ditta G, Schmidhauser T, Yakobson E, Lu P, Liang XW, Finlay DR, Guiney D, Helinski DR. 1985. Plasmids related to the broad host range vector, pRK290, useful for gene cloning and for monitoring gene expression. Plasmid 13:149-53.

2. Sistrom WR. 1960. A requirement for sodium in the growth of *Rhodopseudomonas spheroides.* J Gen Microbiol 22:778-85.

3. Shapleigh JP, Gennis RB. 1992. Cloning, sequencing and deletion from the chromosome of the gene encoding subunit I of the *aa*_3_-type cytochrome *c* oxidase of *Rhodobacter sphaeroides*. Mol Microbiol 6:635-42.

4. Toledo-Cuevas M, Barquera B, Gennis RB, Wikstrom M, Garcia-Horsman JA. 1998. The *cbb*_3_-type cytochrome *c* oxidase from *Rhodobacter sphaeroides,* a proton-pumping heme-copper oxidase. Biochim Biophys Acta 1365:421-34.

5. Swem LR, Kraft BJ, Swem DL, Setterdahl AT, Masuda S, Knaff DB, Zaleski JM, Bauer CE. 2003. Signal transduction by the global regulator RegB is mediated by a redox-active cysteine. EMBO J 22:4699-708.
