# Supplementary material for: Widespread Distribution and Functional Specificity of the Copper Importer CcoA: Distinct Cu Uptake Routes for Bacterial Cytochrome c Oxidases
Source: mBio. 2018 Feb 27;9(1):e00065-18. doi: 10.1128/mBio.00065-18 (PMC5829832; doi:10.1128/mBio.00065-18)
Supplement: TABLE S6 [file mbo001183741st6.docx]

**Table S6.** Primers used in this study.

| **Primer name** | **Primer DNA sequence (5’ to 3’ orientation)** |
| --- | --- |
| CcoAs-BamHI For | GCCGCGGAGGTCGGATCCTGCTGACC |
| CcoAs-XbaI Rev1 | AAGGGCAGCATGTCTAGATTCACCGCCTGCC |
| CcoAs-StuI For | GCGGCGTGGCCCTCTAGGCCTTTCTCGACA |
| CcoAs-StuI Rev | AAGCCCACGCGGAGGCCTAACCGCTGCATG |
| Rsp_2726 ForX | AGCGTCTAGACGGATCTTCGCGGCC |
| Rsp_2726 RevK | TTATGGTACCGGCGGCTTTCACGGC |
| CopAop ForK | AGATGGTACCGCAAGTGCTCACATC |
| CopA RevX | TGCGTCTAGATGAACAACACCGGAT |
| CopAln1F | TCTGGCTCAGGTCATCCAC |
| CopAln1R | AAGGCGGAGAGCAGCGTC |
| CopAln2F | GGAACCGGCGATAGCCGTTC |
| CopAln2R | TGATCGAAGCCACGGCGA |
